# Supplementary material for: The paraventricular thalamus provides a polysynaptic brake on limbic CRF neurons to sex-dependently blunt binge alcohol drinking and avoidance behavior in mice
Source: Nat Commun. 2021 Aug 23;12:5080. doi: 10.1038/s41467-021-25368-y (PMC8382748; doi:10.1038/s41467-021-25368-y)
Supplement: Supplementary file 1 — Supplementary Information [file 41467_2021_25368_MOESM1_ESM.pdf]

Supplementary Figures

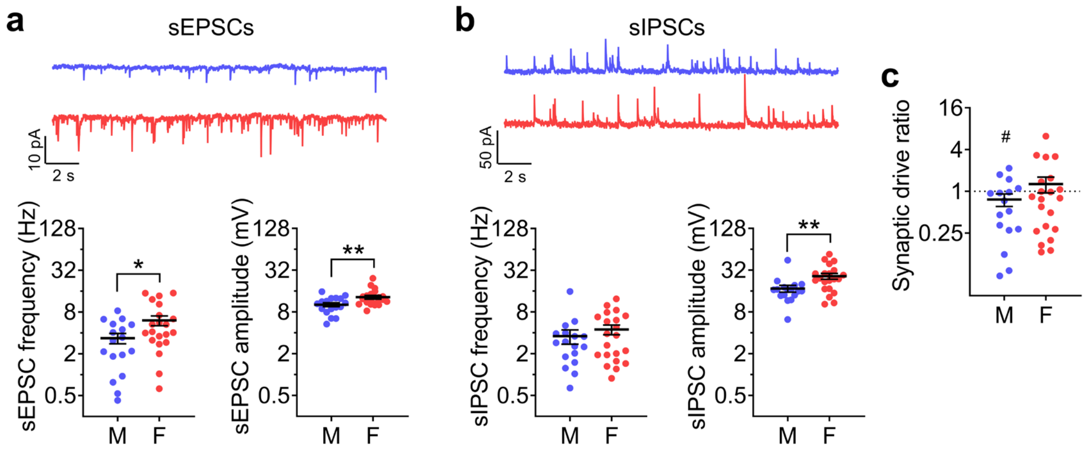

Supplementary Fig. 1: Greater spontaneous glutamate release onto BNST^{CRF} neurons in females than males. Spontaneous excitatory and inhibitory postsynaptic currents (sEPSCs and sIPSCs) in BNST^{CRF} neurons (N's = 8 M, 17 cells; 8 F, 21 cells), analyzed using two-tailed unpaired t-tests. **a,** Top: representative traces of sEPSCs in BNST^{CRF} neurons of males (blue, above) and females (red, below). Bottom: Quantification showing that sEPSC frequency and amplitude are both higher in females than males (frequency, left: t_{36} = 2.20, *P = 0.034; amplitude, right: t_{36} = 2.88, **P = 0.007). **b**, Top: Representative traces of sIPSCs in BNST^{CRF} neurons of males (blue, above) and females (red, below). Bottom: Quantification showing that sIPSC frequency is not different between sexes (left, t_{36} = 0.89, P = 0.380) and amplitude is higher in females (right, t_{36} = 3.66, **P = 0.007). **c,** Synaptic drive ratio, calculated as (sEPSC frequency x amplitude) / (sIPSC frequency x amplitude), in BNST^{CRF} neurons is below 1.0 in males (t_{15} = 2.58, $^{\#}P$ = 0.021) but not females (t_{20} = 1.35, P = 0.191), but there is no difference between males and females (t_{35} = 1.03, P = 0.312). Data are presented as mean values ± SEM. Source data are provided as a Source Data file.

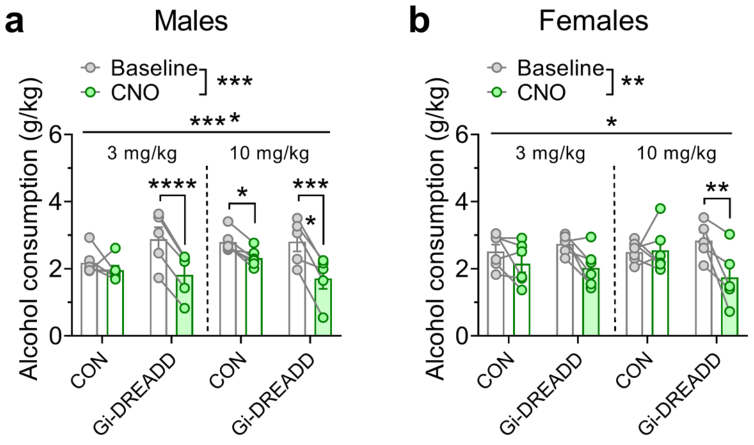

Supplementary Fig. 2: Females require more robust chemogenetic inhibition of BNST^{CRF} neurons to suppress binge alcohol drinking than males. a, Binge alcohol drinking in males is inhibited by both moderate (3 mg/kg, i.p.) and high (10 mg/kg, i.p.) dose clozapine N-oxide (CNO) activation of a Gi-coupled designer receptor exclusively activated by designer drug (Gi-DREADD) in BNST^{CRF} neurons (however, there is a nonspecific effect of high dose CNO in CON males). 3xRM-ANOVA: main effect of CNO ($F_{1,18}$ = 68.18, $^{****}P$ < 0.0001) and CNO x Gi-DREADD interaction ($F_{1,18}$ = 17.72, $^{***}P$ = 0.0005) and no other effects (*P*s > 0.15); post hoc two-tailed paired t-tests with Holm-Sidak corrections between vehicle and CNO within group: CON (3 mg/kg): t_{18} = 1.34, *P* = 0.196; Gi-DREADD (3 mg/kg): t_{18} = 5.86, ****P < 0.0001; CON (10 mg/kg): t_{18} = 2.90, *P = 0.019; Gi-DREADD (10 mg/kg): t_{18} = 6.08, ****P < 0.0001; N's = 6 CON, 5 Gi-DREADD. **b**, Binge alcohol drinking in females is inhibited by high but not moderate dose CNO activation of a Gi-DREADD in BNST^{CRF} neurons. 3xRM-ANOVA: main effect of CNO ($F_{1,19}$ = 14.18, $^{**}P$ = 0.001) and CNO x Gi-DREADD interaction ($F_{1,19}$ = 6.96, $^{*}P$ = 0.016) but no other effects (*P*s > 0.15); post hoc two-tailed paired t-tests with Holm-Sidak corrections between vehicle and CNO within group: CON (3 mg/kg): t_{19} = 1.34, *P* = 0.355; Gi-DREADD (3 mg/kg): t_{18} = 2.59, *P* = 0.053; CON (10 mg/kg): t_{18} = 0.18, *P* = 0.857; Gi-DREADD (10 mg/kg): t_{18} = 3.63, **P = 0.007; N's = 6 CON, 6 Gi-DREADD. Data are presented as mean values ± SEM. Source data are provided as a Source Data file. (3 mg/kg data are adapted from Pleil et al., 2015 and here represented separately by sex.)

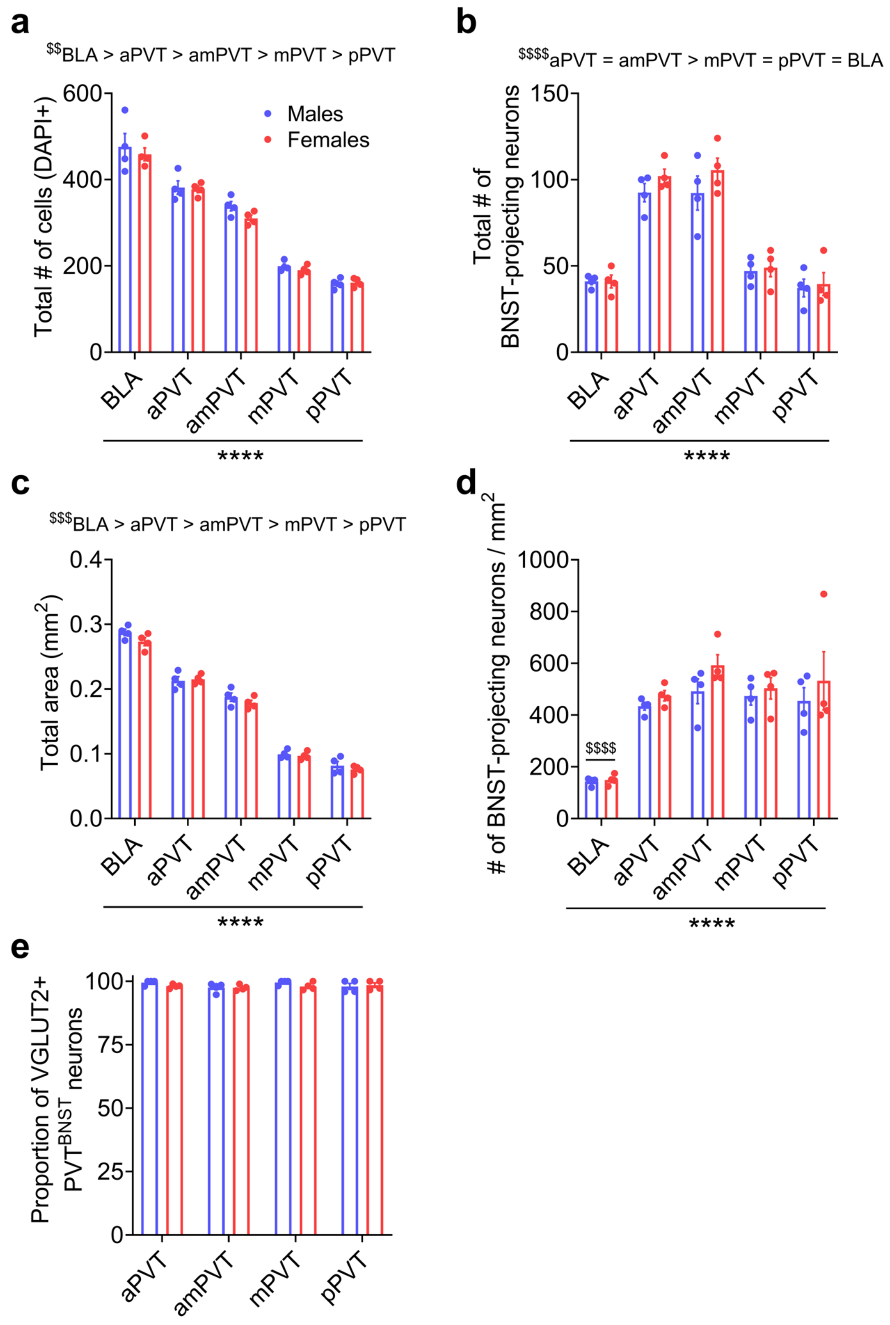

Supplementary Fig. 3: Detailed quantification of glutamatergic inputs to the BNST (related to Fig. 2). a, Total number of cells per region/subregion in both sexes, indicated by a DAPI counterstain of nuclei. Mixed-effects model: main effect of subregion ($F_{4,23}$ = 324.0, ****P < 0.0001) but no effect of sex (P = 0.284) or interaction (P = 0.600), with BLA>aPVT>amPVT>mPVT>pPVT according to post hoc two-tailed t-tests with Holm-Sidak corrections ($^{\$\$\$\$}P$s < 0.0001 for all direct subregion comparisons except mPVT vs. pPVT $^{\$\$}P$ = 0.002). **b,** Total raw number of BNST-projecting neurons across subregions. Mixed-effects model: main effect of subregion ($F_{4,23}$ = 111.1, ****P < 0.0001) but no effect of sex (P = 0.331) or interaction (P = 0.556), with aPVT=amPVT>mPVT=pPVT=BLA according to post hoc two-tailed t-tests with Holm-Sidak corrections. $^{\$\$\$\$}P$s < 0.0001 for all significantly different subregion comparisons; others: aPVT vs. amPVT (P = 0.908), BLA vs. mPVT (P = 0.168), BLA vs. pPVT (P = 0.908), mPVT vs. pPVT (P = 0.109). **c,** Total area of each subregion. Mixed-effects model: main effect of subregion ($F_{4,30}$ = 559.9, ****P < 0.0001) but no effect of sex (P = 0.069) or interaction (P = 0.534), with BLA>aPVT>amPVT>mPVT>pPVT according to post hoc two-tailed t-tests with Holm-Sidak corrections ($^{\$\$\$\$}P$s < 0.0001 for all direct subregion comparisons except mPVT vs. pPVT $^{\$\$\$}P$ = 0.0006). **d,** Number of BNST-projecting neurons normalized to subregion area. Mixed-effects model: main effect of subregion ($F_{4,23}$ = 28.83, ****P < 0.0001) but no effect of sex (P = 0.234) or interaction (P = 0.855), with post hoc two-tailed t-tests with Holm-Sidak corrections showing BLA<all PVT subregions ($^{\$\$\$\$}P$s < 0.0001); others: aPVT vs. amPVT (P = 0.249), vs. mPVT (P = 0.735), vs. pPVT (P = 0.735); amPVT vs. mPVT

(P = 0.697), vs. pPVT (P = 0.697); mPVT vs. pPVT (P = 0.907). **e,** Proportion of PVT^{BNST} neurons that are VGLUT2+. 2xRM-ANOVA shows no effects of sex (P = 0.297) or subregion (P = 0.289) or interaction (P = 0.418). Data are presented as mean values ± SEM. Source data are provided as a Source Data file.

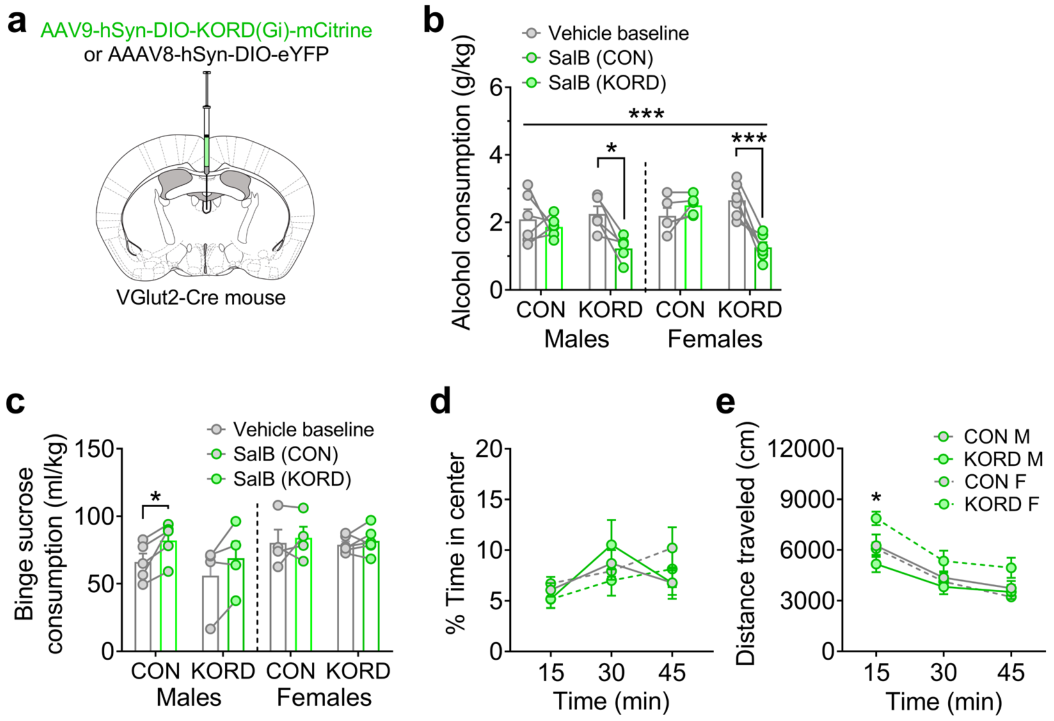

Supplementary Fig. 4: PVT glutamate neurons modulate binge drinking in both sexes. a, Inhibitory Gi-coupled KOR DREADD (KORD) strategy in PVT^{VGLUT2} neurons. **b,** One-hr alcohol consumption during within-cycle vehicle baseline and Salvinorin B (SalB, 17 mg/kg, s.c.) on Day 4 (Ns = 6 CON M, 5 Gi-KORD M, 5 CON F, 6 Gi-KORD F). 3xRM-ANOVA: main effects of the KORD ($F_{1,18}$ = 4.92, *P* = 0.037) and SalB ($F_{1,18}$ = 16.17, *P* = 0.0008), and a KORD x SalB interaction ($F_{1,18}$ = 18.51, ****P* = 0.0004); post hoc two-tailed paired t-tests with Holm-Sidak correction for effect of SalB: Gi-KORD M (t_4 = 3.79, **P* = 0.019), Gi-KORD F(t_5 = 5.29, ****P* = 0.0003), CON M and F groups (*P*s > 0.15). **c,** Sucrose consumption during 4% sucrose DID (Ns = 5 CON M, 4 Gi-KORD M, 4 CON F, 6 Gi-KORD F after 0-1 mouse/group excluded for no baseline consumption). 3xRM-ANOVA: main effect of SalB ($F_{1,15}$ = 11.88, *P* = 0.004) and a SalB x sex interaction ($F_{1,15}$ = 4.79, *P* = 0.045) but no effect of the KORD or sex or other interactions (*P*s > 0.05); post hoc two-tailed paired t-tests with Holm-Sidak corrections on the effect of SalB: CON males (t_4 = 3.54, **P* = 0.024; all others *P* > 0.10). **d-e,** Effects of SalB activation of the Gi-KORD on OF behavior. Ns = 6 CON M and 5 in other groups. **d,** Percent time in center. 3xRM-ANOVA: main effect of time ($F_{1.9,32.4}$ = 6.05, *P* = 0.007) and a sex x time interaction ($F_{2,34}$ = 3.69, *P* = 0.035) but no other effects (*P*s > 0.35); post hoc two-tailed unpaired t-tests with Holm-Sidak corrections within each 15 min bin (*P*s > 0.35). **e,** Distance traveled. 3xRM-ANOVA: main effect of time ($F_{1.7,28.8}$ = 116.6, *P* < 0.0001, not indicated) and a sex x KORD interaction ($F_{1,17}$ = 7.06, *P* = 0.017) but no other effects (*P*s > 0.05); post hoc two-tailed t-tests with Holm-Sidak corrections: 0-15 min CON vs. Gi-KORD F (t_8 = 3.28, **P* = 0.033; all other comparisons *P*s > 0.05). Data are presented as mean values $\pm$ SEM. Source data are provided as a Source Data file.

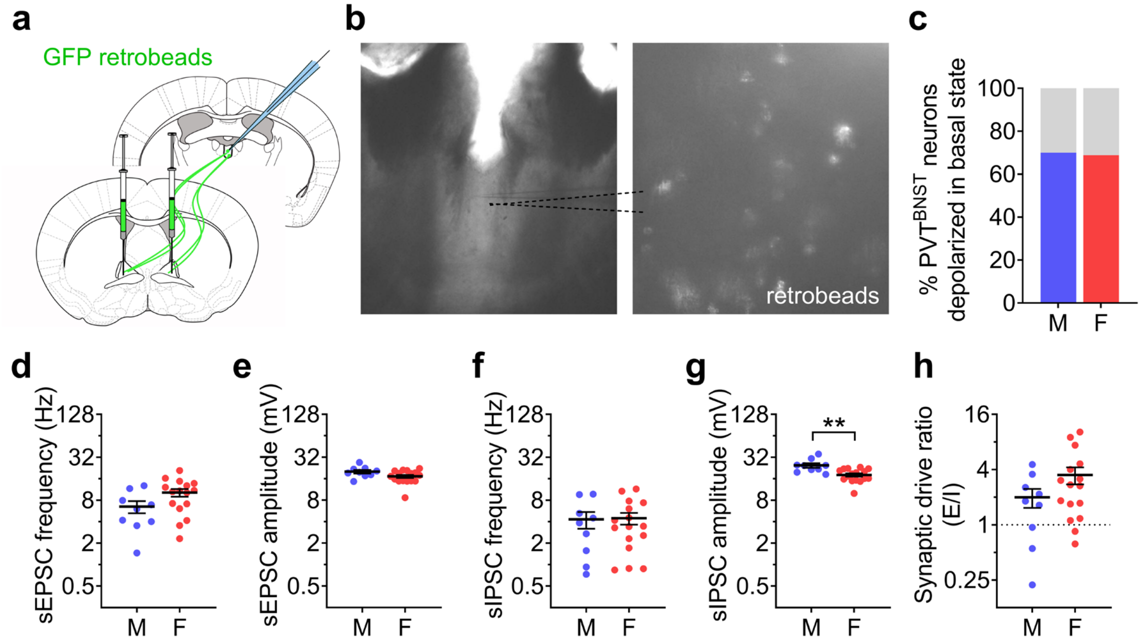

Supplementary Fig. 5: Electrophysiological characterization of PVT^{BNST} neurons. a, Retrograde labeling strategy used to identify and perform electrophysiology recordings in BNST-projecting PVT (PVT^{BNST}) neurons. **b,** Representative image of a coronal PVT brain slice at 4x (left) containing GFP retrobead-positive cell bodies shown magnified at 40x (right) using monochrome camera on the slice electrophysiology rig. **c,** Proportion of sampled PVT^{BNST} neurons active in their basal state is not different between males and females (Fisher's exact test: $P > 0.999$; N's = 8 M, 20 cells; 6 F, 19 cells). **d-h**, Synaptic transmission measures from PVT^{BNST} neurons (N's = 4 M, 9 cells; 5 F, 16 cells; two-tailed unpaired t-tests). **d,** sEPSC frequency ($t_{23} = 1.87$, $P = 0.075$). **e,** sEPSC amplitude ($t_{23} = 1.86$, $P = 0.075$). **f,** sIPSC frequency ($t_{23} = 0.20$, $P = 0.840$). **g,** sIPSC amplitude ($t_{23} = 3.45$, $^{**}P = 0.002$). **h,** Synaptic drive ratio ($t_{22} = 0.79$, $P = 0.438$ with Welch's correction). Data are presented as mean values $\pm$ SEM. Source data are provided as a Source Data file.

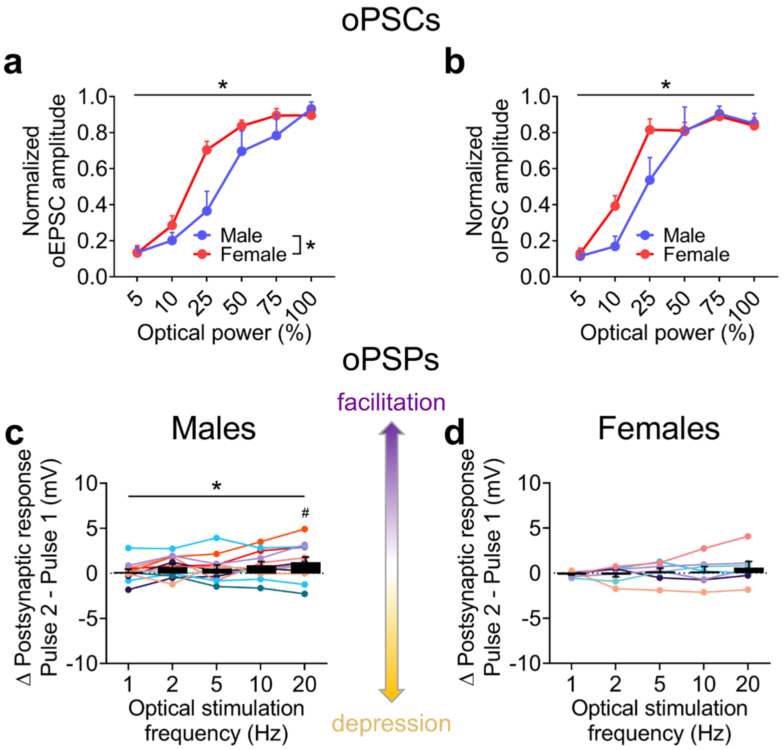

Supplementary Fig. 6: Additional characterization of PVT-BNST^{CRF} synapses (related to Fig. 3). a-b, oEPSC (**a**) and oIPSC (**b**) amplitude across LED power (**Fig. 3e,f**) normalized to the maximum response within each cell shows that females have a left-shifted curves compared to males. **a,** oEPSCs. 2xRM-ANOVA: main effect of sex ($F_{1,17}$ = 6.18, *P = 0.024) and power ($F_{5,85}$ = 65.0, P < 0.0001, not indicated) and a sex x time interaction ($F_{5,85}$ = 2.69, *P = 0.026), with no significant post hoc two-tailed unpaired t-tests with Holm-Sidak corrections within LED power (Ps > 0.10). **b,** oIPSCs. 2xRM-ANOVA: main effect of time ($F_{5,85}$ = 65.0, P < 0.0001, not indicated) and a sex x time interaction ($F_{5,85}$ = 2.41, *P = 0.043), as well as a trend of sex ($F_{1,17}$ = 4.30, P = 0.054), with no significant post hoc two-tailed unpaired t-tests with Holm-Sidak corrections within LED power (Ps > 0.05). **c-d,** Differences between the oPSP elicited from the second and first pulses of LED stimulation across a range of stimulation frequencies within individual BNST^{CRF} neurons during current-clamp recordings, with positive delta values indicating facilitated responses and negative delta values indicating depressed responses (N's = 7 M, 12 cells; 6 F, 9 cells). **c,** In males, oPSPs from second pulses are larger than first pulses at 20 Hz stimulation but for no other frequencies. 1xRM-ANOVA effect of frequency ($F_{4,44}$ = 2.61, *P = 0.025), with one-sample t-tests for delta values at each frequency against the null hypothesis value of 0 showing a significant facilitation at 20 Hz (t_{11} = 2.23, $^{\#}P$ = 0.048) but no other frequencies (Ps > 0.05). **d,** In females, oPSPs from first and second pulses of stimulation are similar across all frequencies (1xRM-ANOVA no effect of frequency ($F_{4,24}$ = 0.77, P > 0.55), and no significant one-sample t-tests within frequency (Ps > 0.35). Data are presented as mean values + SEM. Source data are provided as a Source Data file.

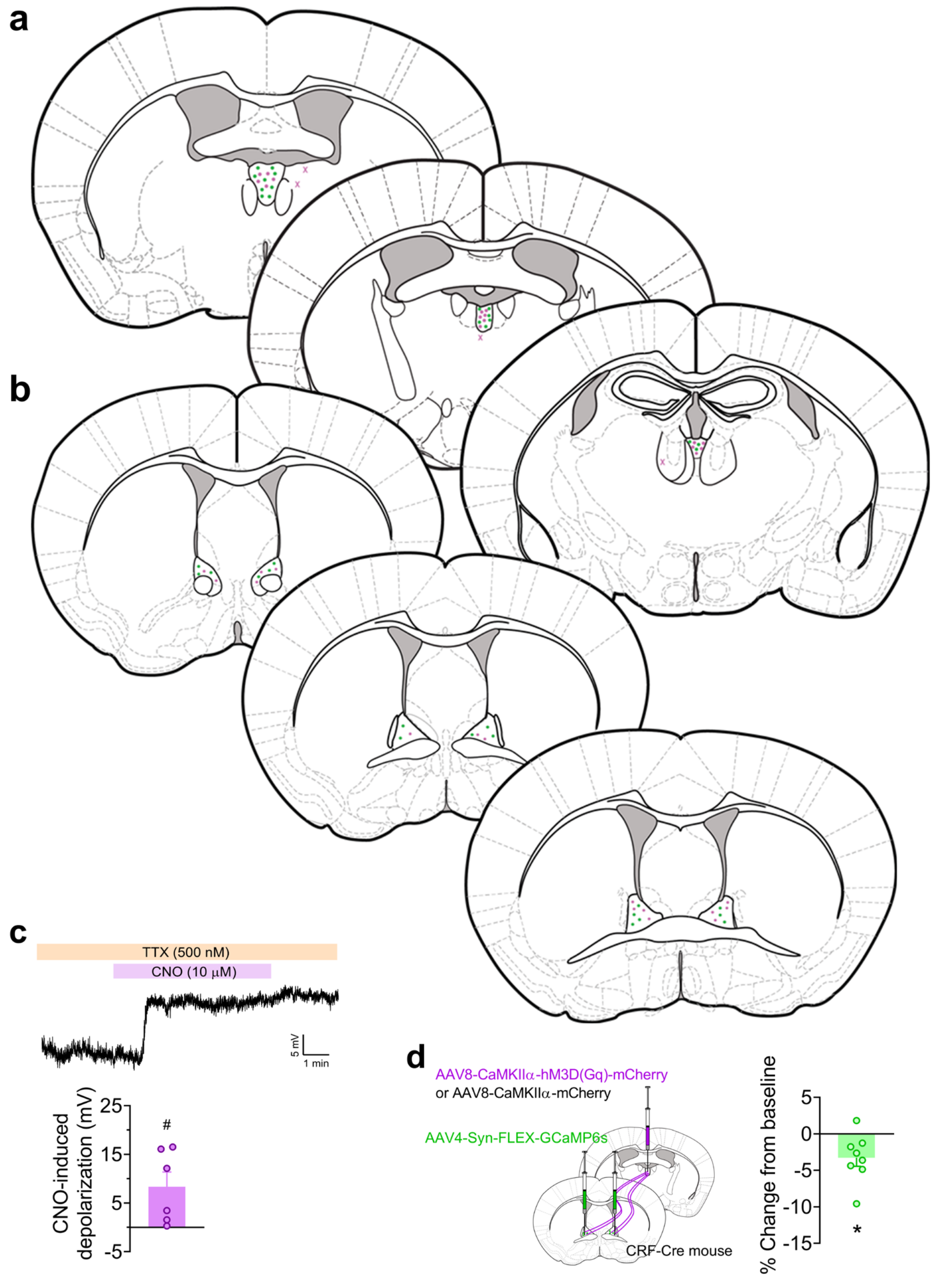

Supplementary Fig. 7: Additional measures of DREADD activation on physiology (related to Fig. 5). a-b, Viral injection placements in PVT (**a**) and BNST (**b**) for DREADD mice, with hits indicated with circles and misses excluded from analysis in X's (CONs in green, DREADDs in pink). **c,** Slice electrophysiology in a subset of DREADD mice shows that in the presence of tetrodotoxin (TTX) to block action potentials, bath application of clozapine N-oxide (CNO, 10 μM) depolarizes DREADD+ PVT cell bodies, as shown in the trace above and quantified in the bar graph below (one-sample t-test compared to 0 mV change: $t_5 = 2.75$, $^{\#}P = 0.040$). N = 3 mice, 6 cells. **d,** left: Schematic of the strategy to express the calcium biosensor GCaMP6s specifically in $BNST^{CRF}$ neurons and the Gq-DREADD or empty control virus in the PVT for *ex vivo* slice calcium imaging experiment in the BNST during bath application of CNO (10 μM) to activate the Gq-DREADD in PVT terminals. Right: Quantified percent change in fluorescence intensity of GCaMP6s+ in $BNST^{CRF}$ neurons in the presence of CNO from baseline in mice with the Gq-DREADD in the PVT (normalized to background image intensity and standard linear decay of the fluorescence signal observed in neurons from CON virus mice). One-sample t-test: $t_7 = 2.82$, $^{*}P = 0.026$. N's = 1 CON, 6 cells; 2 Gq-DREADD, 8 cells. Data are presented as mean values $\pm$ SEM. Source data are provided as a Source Data file.

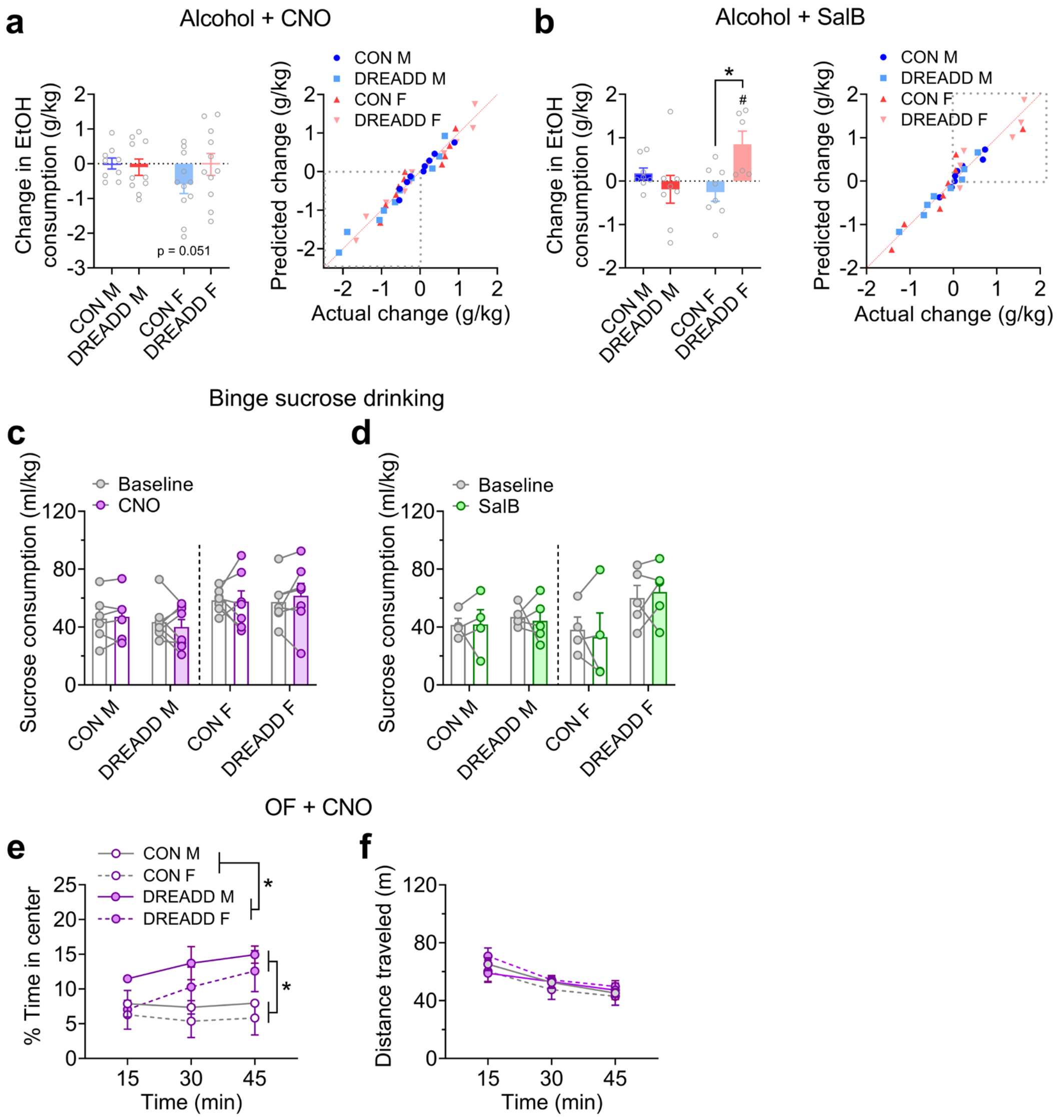

Supplementary Fig. 8: Additional measures of DREADD activation on behavior (related to Fig. 5). a-b, Complementary analyses for DID alcohol consumption in **Fig. 5c,d**. **a,** Left: two-tailed unpaired t-tests between CON and DREADD mice within sex comparing the CNO-induced change in alcohol drinking show no differences (*P*s > 0.20), but DREADD M show a nearly-significant decrease from baseline (one-sample t-test: t_{10} = 2.18, *P* = 0.055), while other groups do not (*P*s > 0.65). Right: QQ plots comparing actual change to predicted change in alcohol consumption, with DREADD M data primarily represented in lower left quadrant (boxed area). **b**, Left: DREADD F have a significant SalB-induced increase in binge drinking (one-sample t-test: t_5 = 2.83, $^{\#}P$ = 0.037; all other *P*s > 0.20); this change is greater than CON F (two-tailed unpaired t-test: t_{26} = 2.85, **P* = 0.017). Right: QQ plots comparing actual change to predicted change in alcohol consumption, with DREADD F data primarily represented in upper right quadrant (boxed area). **c-d,** DREADD activation does not affect two-hr binge sucrose consumption. **c,** CNO administration. 3xRM-ANOVA: main effect of sex ($F_{1,24}$ = 6.77, *P* = 0.016, not indicated) but no other effects or interactions (*P*s > 0.30). N's = 6 CON M, 8 DREADD M, 7 CON F, 7 DREADD F. **d,** SalB administration. 3xRM-ANOVA: no effects or interactions (*P*s > 0.05). N's = 4 CON M, 5 DREADD M, 4 CON F, 5 DREADD F. **e-f,** CNO administration decreases avoidance of the center of the open field (OF) in DREADD mice without altering locomotion, confirming the anxiolytic effect in the EPM in **Fig. 5e-g**. **e,** 3xRM-ANOVA on percent time in center: main effects of DREADD ($F_{1,7}$ = 8.39, **P* = 0.023) and time ($F_{1.9,13.5}$ = 4.18, *P* = 0.040, not indicated) and a DREADD x time interaction ($F_{2,14}$ = 5.33, **P* = 0.019). **f,** 3xRM-ANOVA on the distance traveled shows a main effect of time ($F_{1.5,10.3}$ = 56.18, *P* < 0.0001, not indicated), but no other effects or interactions (*P*s > 0.15). N's = 3 CON M, 3 DREADD M, 3 CON F, 2 DREADD F. Data are presented as mean values ± SEM. Source data are provided as a Source Data file.

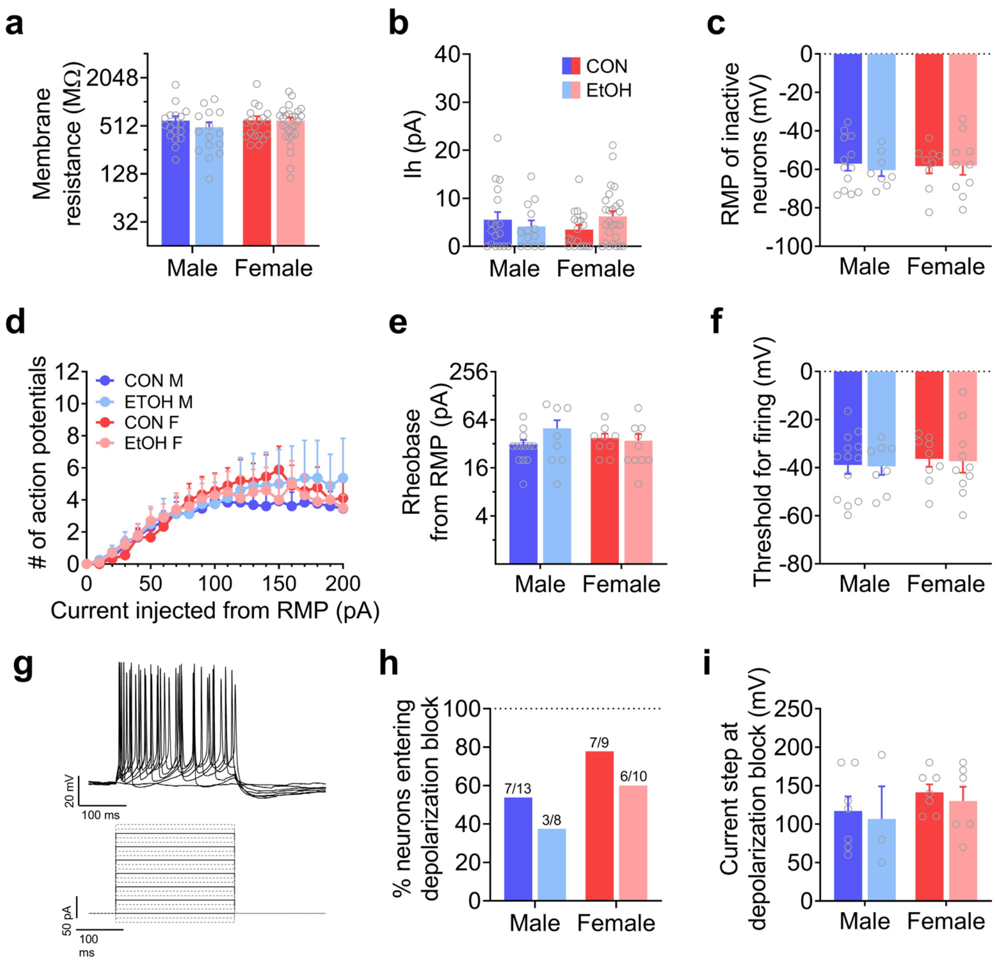

Supplementary Fig. 9: Effects of sex and alcohol on neuronal excitability in BNST^{CRF} neurons at RMP (related to Fig. 6b). a, Membrane resistance measured from a current-voltage (I-V) plot with hyperpolarizing steps in voltage-clamp. 2xANOVA: no effects of or interaction between sex and EtOH (*P*s > 0.25). **b,** Hyperpolarization-activated depolarizing current (I_h). 2xANOVA: no effects or interaction between sex and EtOH (*P*s > 0.10). **c,** Resting membrane potential (RMP) of neurons that were basally inactive. 2xANOVA: no effects or interaction between sex and EtOH (*P*s > 0.65). **d-h**, Measures from a V-I plot in current-clamp with increasing 10 pA steps of current injection (from -20 pA to 200 pA) into basally inactive cells from their RMP (cells identified and represented in **c**). **d**, Number of action potentials generated across current steps. 3xRM-ANOVA: main effect of current step ($F_{20,720}$ = 25.57, *P* < 0.0001, not indicated) but no other effects or interactions (*P*s > 0.60). **e,** Rheobase (minimum amount of current required to elicit an action potential). 2xANOVA: no effects or interaction between sex and EtOH (*P*s > 0.20). **f**, Voltage threshold for firing. 2xANOVA: no effects or interactions (*P*s > 0.55). **g**, Representative trace (above) and stimulus protocol with sweeps represented by alternating solid and dashed lines (below) for V-I plot. **h-i**, Proportion of neurons that enter depolarization block within the V-I plot is not different between sex or affected by EtOH (Fisher's exact tests: *P*s > 0.35) (**h**) and the step at which this occurs is unaffected (**i**; 2xANOVA: no effects or interaction (*P*s > 0.25)). N's = 9 CON M, 18 cells; 6 EtOH M, 15 cells; 9 CON F, 20 cells; 11 EtOH F, 27 cells. Tonically active cells were excluded from analysis for measures in **c-i**. Data are presented as mean values + SEM. Source data are provided as a Source Data file.

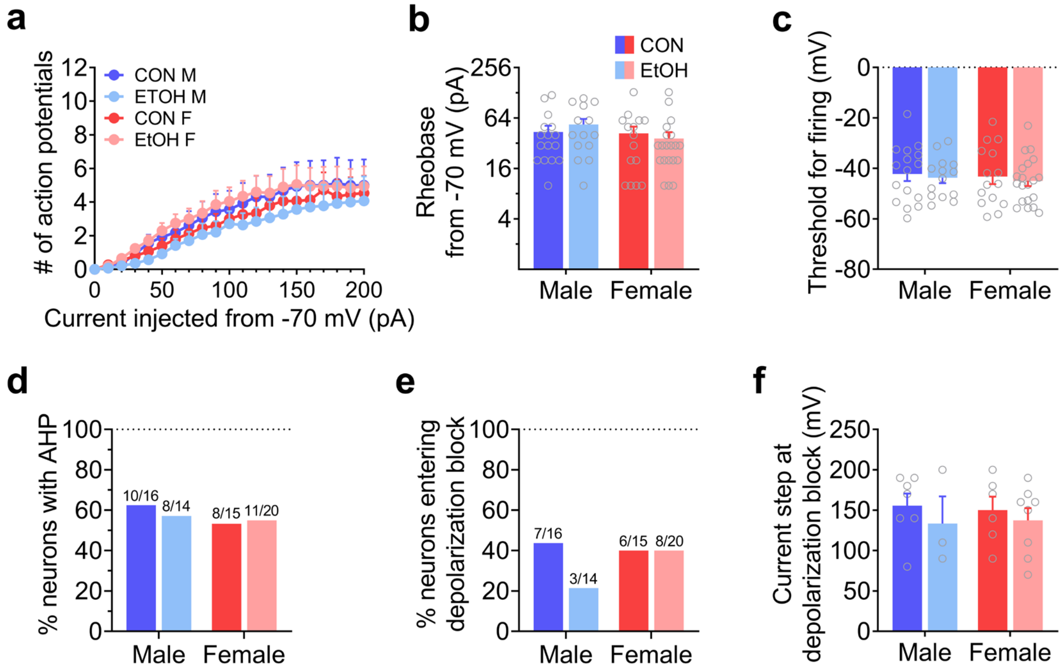

Supplementary Fig. 10: Effects of sex and alcohol on neuronal excitability in BNST^{CRF} neurons when held at a common membrane potential of -70 mV. a, Number of action potentials generated across current steps during a V-I plot starting at a holding potential of -70 mV in all neurons except those displaying firing at this potential (same as **Supplementary Fig. 9d-i** but starting from -70 mV holding potential). 3xRM-ANOVA: main effect of current step ($F_{20,1220}$ = 38.32, $P < 0.0001$, not indicated) but no other effects of or interactions between sex, EtOH, and step (*P*s > 0.25). **b,** Rheobase. 2xANOVA: no effects or interaction (*P*s > 0.10). **c,** Voltage threshold for firing. 2xANOVA: no effects or interaction (*P*s > 0.50). **d,** Percentage of neurons displaying an after-hyperpolarization potential (AHP) following action potentials elicited by current injection during the V-I plot is not different between sex or affected by EtOH (Fisher's exact tests: *P*s > 0.70). **e-f**, Proportion of neurons that enter depolarization block within the V-I plot is not different between sex or affected by EtOH (Fisher's exact tests: *P*s > 0.25). (**e**) and the step at which this occurs is unaffected (**f**; 2xANOVA: no effects or interaction (*P*s > 0.35)). The same lack of effects is observed when a ramp current injection protocol is employed (unpublished data). Ns are the same as **Supplementary Fig. 9** prior to exclusion based on firing activity at -70 mV. Data are presented as mean values + SEM. Source data are provided as a Source Data file.

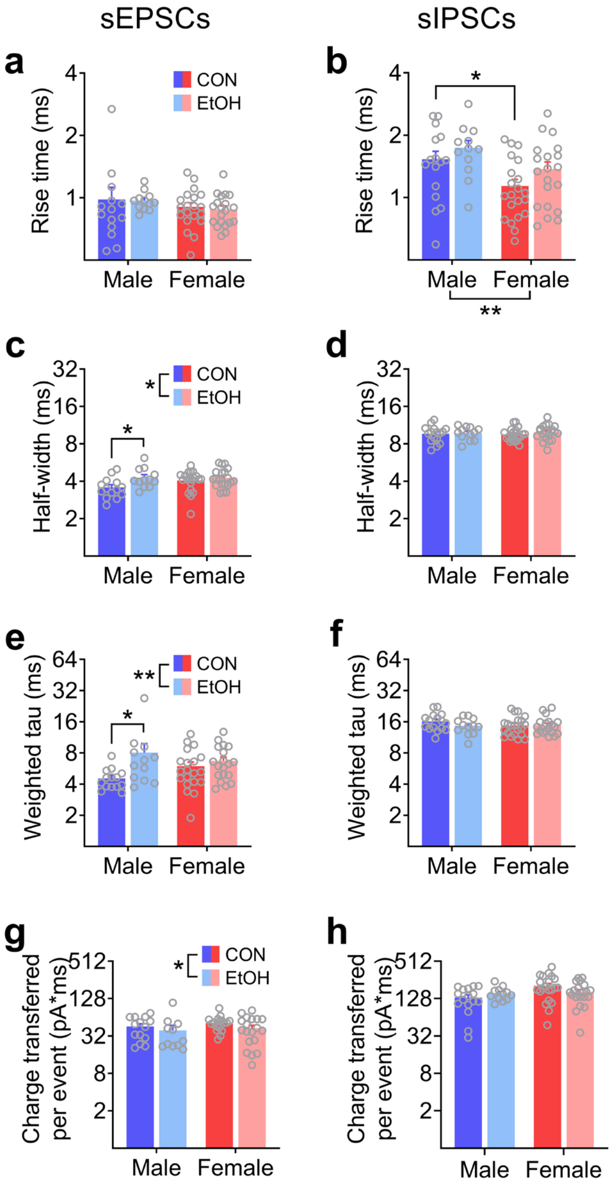

Supplementary Fig. 11: Sex differences and alcohol-induced plasticity in spontaneous postsynaptic current (sPSC) kinetics in BNST^{CRF} neurons (related to Fig. 6c-h). a, sEPSC rise time. 2xANOVA: no effects of or interaction between sex and EtOH (*P*s > 0.30). **b,** sIPSC rise time. 2xANOVA: main effect of sex ($F_{1,65}$ = 9.59, ***P* = 0.003) but no other effects (*P*s > 0.05), with a post hoc two-tailed unpaired t-test confirming a difference between CON males and females (t_{65} = 2.37, **P* = 0.021). **c,** sEPSC half-width. 2xANOVA: main effect of alcohol ($F_{1,62}$ = 6.48, **P* = 0.013) but no other effects (*P*s > 0.15); post hoc two-tailed unpaired t-tests with Holm-Sidak corrections show an effect of alcohol in males (t_{62} = 2.52, **P* = 0.028) but not females (*P* = 0.358). **d,** sIPSC half-width. 2xANOVA: no effects or interaction (*P*s > 0.40). **e,** sEPSC weighted tau. 2xANOVA: main effect of EtOH ($F_{1,61}$ = 7.94, ***P* = 0.007) but no other effects (*P*s > 0.15); post hoc two-tailed unpaired t-tests with Holm-Sidak corrections show the effect of EtOH in males (t_{61} = 2.72, **P* = 0.017) but not in females (*P* = 0.266). **f,** sIPSC weighted tau. 2xANOVA: no effects or interaction (*P*s > 0.25). **g,** sEPSC charge transferred per event, calculated as average area per sEPSC. 2xANOVA: main effect of alcohol ($F_{1,57}$ = 4.04, **P* = 0.049) but no other effects (*P*s > 0.25); post hoc two-tailed unpaired t-tests with Holm-Sidak corrections show the effect of alcohol was not driven by either sex (*P*s > 0.10). **h,** sIPSC charge transferred per event. 2xANOVA: no effects or interaction (*P*s > 0.05). For all analyses, cells with an average amplitude of 5 pA or lower after filtering were excluded from analysis—for sEPSCs: 3 CON M, 1 EtOH M, 1 EtOH F; for sIPSCs: 1 CON M, 1 EtOH F. Data are presented as mean values + SEM. Source data are provided as a Source Data file.

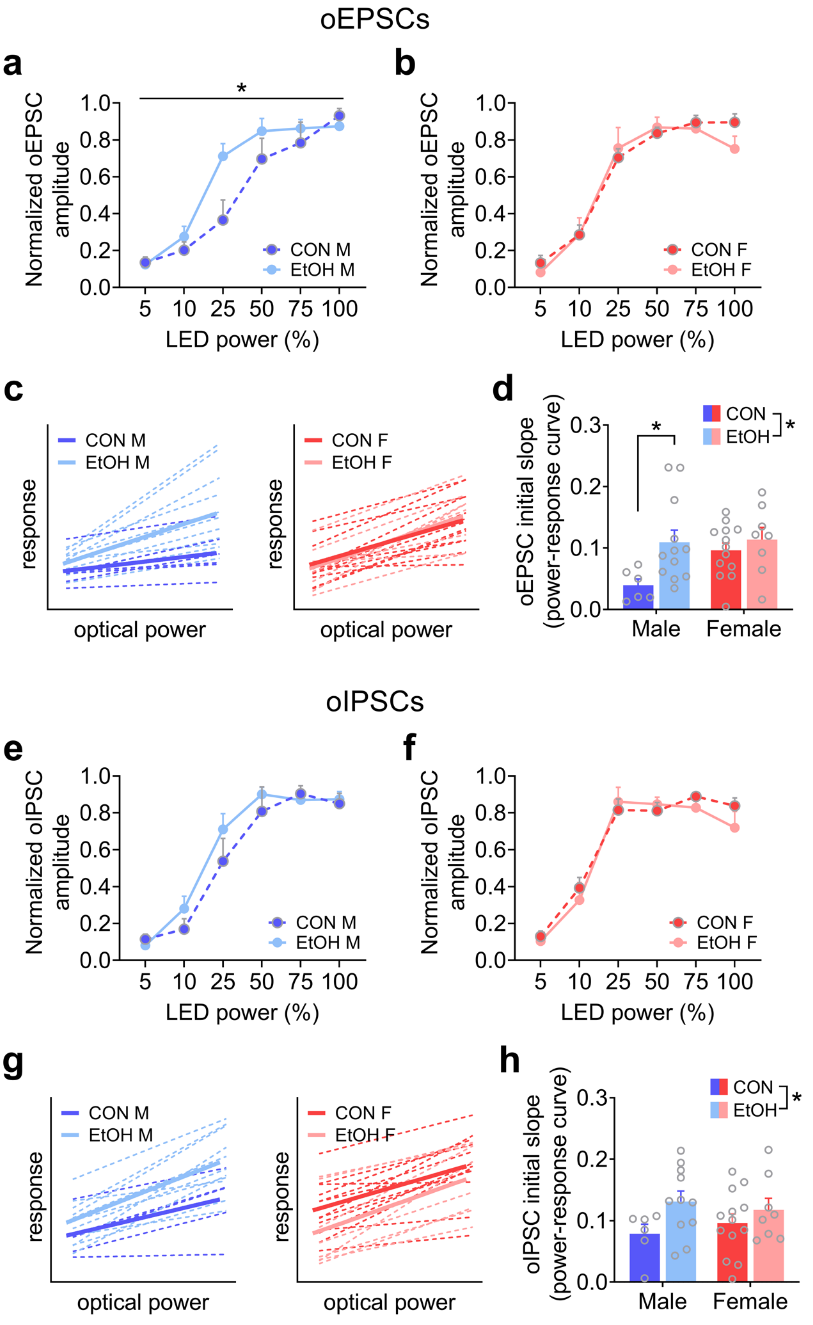

Supplementary Fig. 12: Complementary analyses of PVT-evoked oPSCs (related to Fig. 7). a-b, oEPSC amplitude across LED power normalized to the maximum response within cell, measured with 2xRM-ANOVAs. **a,** Leftward shift in EtOH M (increased amplitude at lower optical power) compared to CON M: EtOH x time interaction ($F_{5,80}$ = 2.50, **P* = 0.037) and a main effect of power ($F_{5,80}$ = 51.57, *P* < 0.0001, not indicated) but no main effect of EtOH (*P* > 0.10), and no significant differences in post hoc two-tailed unpaired t-tests with Holm-Sidak corrections within LED power (*P*s > 0.10). **b,** No effects of EtOH in females: main effect of power ($F_{5,95}$ = 73.98, *P* < 0.0001, not indicated) but no other effects (*P* > 0.50). **c,** Linear regression for PVT-evoked oEPSCs in BNST^{CRF} neurons across 5-25% LED power of (males: left, females: right), with dashed lines showing fits for individual cells and solid lines showing group means. Regression equations: CON M (Y=0.03946*X+1.462), EtOH M (Y=0.1095*X+1.438), CON F (Y= 0.09632*X+1.461), EtOH F (Y=0.1137*X+1.209). **d,** oEPSC slope calculated in **c**. 2xANOVA: main effect of EtOH ($F_{1,35}$ = 6.01, **P* = 0.019) but no effect of sex or interaction (*P*s > 0.05); post hoc two-tailed unpaired t-tests with Holm-Sidak corrections show that EtOH was driven by males (M: t_{35} = 2.64, **P* = 0.025; F: *P* > 0.45). **e-h,** same as **a-d** but for oIPSCs. **e,** Males: main effect of power ($F_{5,80}$ = 57.73, *P* < 0.0001, not indicated) but no other effects (*P* > 0.25). **f,** Females: main effect of power ($F_{5,95}$ = 62.69, *P* < 0.0001, not indicated) but no other effects (*P* > 0.35). **g,** Regression equations: CON M (Y=0.07869*X+1.624), EtOH M (Y=0.1312*X+1.926), CON F (Y=0.09628*X+2.634), EtOH F

(Y=0.1178*X+1.523). **h,** oIPSC slope calculated in **g**. 2xANOVA: main effect of EtOH ($F_{1,34}$ = 4.42, *P = 0.043) but no effect of sex or interaction (*P*s > 0.35); no differences in post hoc two-tailed unpaired t-tests with Holm-Sidak corrections (*P* > 0.10). Data in **c** and **g** and presented as mean values; others are mean + SEM. Source data are provided as a Source Data file.

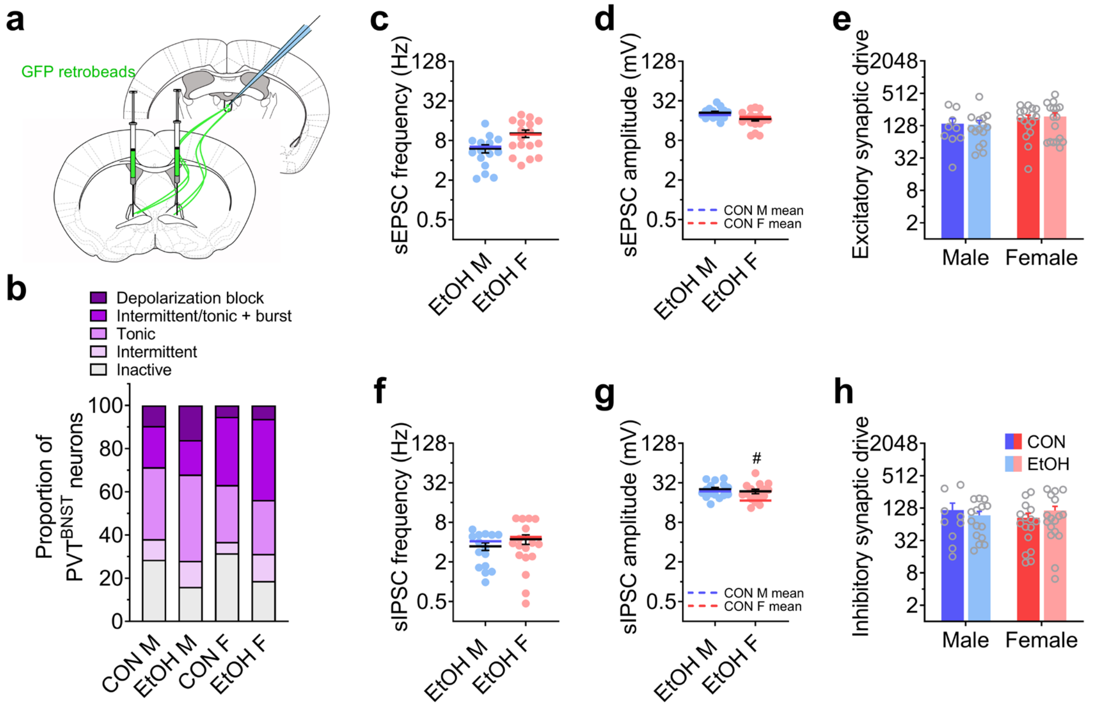

Supplementary Fig. 13: Effects of sex and chronic alcohol on synaptic transmission in PVT^{BNST} neurons. a, Retrograde labeling of PVT^{BNST} neurons for electrophysiology recordings one day following 3-cycle DID. **b, %** PVT^{BNST} neurons in various states of excitability, with no effect of EtOH in either sex (Fisher's exact test, *P*s > 0.45). Ns = 8 EtOH M, 25 cells; 6 EtOH F, 16 cells. **c-d**, spontaneous excitatory postsynaptic current (sEPSC) frequency (**c**) and amplitude (**d**) in PVT^{BNST} neurons in EtOH M and EtOH F mice (with blue and red lines indicating the means in water CON M and F from **Supplementary Fig. 5**). Two-tailed unpaired t-tests evaluating the effect of EtOH within sex for frequency (M: t_{22} = 0.14, *P* = 0.887; F: t_{31} = 0.13, *P* = 0.990) and amplitude (M: t_{22} = 0.58, *P* = 0.565; F: t_{31} = 0.32, *P* = 0.753). **e,** Excitatory synaptic drive, calculated as sEPSC frequency x sEPSC amplitude, within individual neurons. 2xANOVA: no effects (*P*s > 0.55). **f-g**, spontaneous inhibitory postsynaptic current (sIPSC) frequency (**f**) and amplitude (**g**) in the same PVT^{BNST} neurons in **c** and **d**. Two-tailed unpaired t-tests evaluating the effect of EtOH within sex for frequency (M: t_{22} = 0.111, *P* = 0.912; F: t_{31} = 0.011, *P* = 0.991) and amplitude (M: t_{22} = 0.175, *P* = 0.863; F: t_{31} = 2.69, $^{\#}P$ = 0.011). **h**, Inhibitory synaptic drive within individual neurons. 2xANOVA: no effects (*P*s > 0.70). For **c-h**, N's = 5 EtOH M, 15 cells; 6 EtOH F, 17 cells. Data are presented as mean values ± SEM. Source data are provided as a Source Data file.

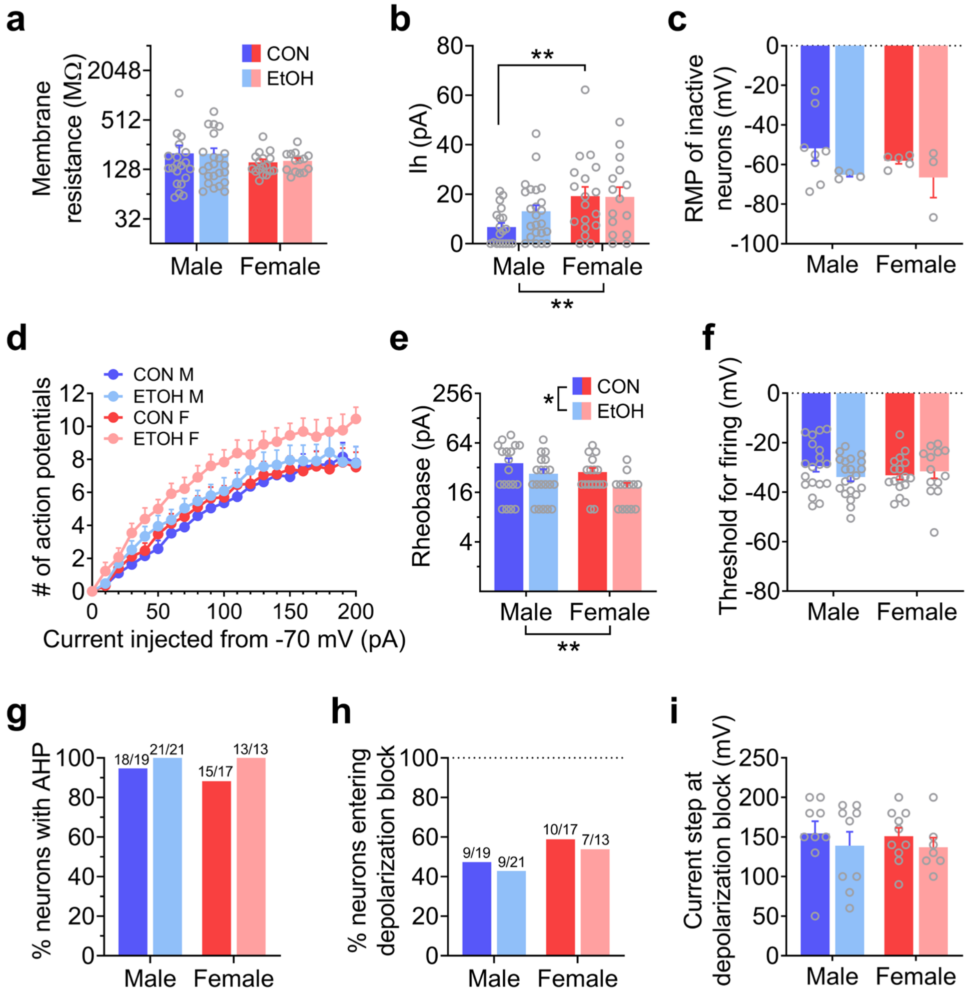

Supplementary Fig. 14: Effects of sex and alcohol on neuronal excitability in PVT^{BNST} neurons. a, Membrane resistance measured from a current-voltage (I-V) plot in voltage clamp configuration with hyperpolarizing steps. 2xANOVA: no effects (*P*s > 0.80). **b,** Hyperpolarization-activated depolarizing current (Ih) measured in the I-V plot. 2xANOVA: main effect of sex ($F_{1,73}$ = 10.19, ***P* = 0.002); a post hoc two-tailed unpaired t-test confirms a sex difference between CON M and F (t_{73} = 3.11, ***P* = 0.003). **c,** Resting membrane potential (RMP) of basally inactive neurons. 2xANOVA: no effects (*P*s > 0.10). **d-f**, Measures from a V-I plot in current clamp in with increasing 10 pA steps of current injection in all neurons inactive at this potential, starting at a holding potential of -70 mV. N's = 8 CON M, 21 cells; 8 EtOH M, 25 cells; 6 CON F, 19 cells; 6 EtOH F, 16 cells. **d**, Number of action potentials generated across current steps. 3xRM-ANOVA: main effect of current step ($F_{2.5,\ 164}$ = 158.8, *P* < 0.0001) and trend of EtOH ($F_{1,66}$= 3.87, *P* = 0.054) but no other effects (*P*s > 0.15). **e,** Rheobase. 2xANOVA: main effect of EtOH ($F_{1,65}$ = 5.32, **P* = 0.024) and main effect of sex ($F_{1,65}$ = 4.03, **P* = 0.049) but no interaction (*P* > 0.95), with post hoc two-tailed t-tests showing the effect of alcohol was not driven by one sex (*P*s > 0.15). **f**, Voltage threshold for firing. 2xANOVA: no effects (*P*s > 0.10). **g,** Proportion of neurons displaying an after-hyperpolarization potential (AHP) following spontaneous action potentials is not different between sex or affected by EtOH (Fisher's exact test *P*s > 0.45; N's = 8 CON M, 19 cells; 6 CON F, 17 cells; 8 EtOH M, 19 cells; 6 EtOH F, 13 cells). **h-i**, Proportion of neurons that enter depolarization block within the V-I plot is not different between sex or affected by EtOH (Fisher's exact test *P*s > 0.50) (**h**) and the step at which this occurs is unaffected (**i**; 2xANOVA: no effects (*P*s > 0.30)). Data are presented as mean values + SEM. All source data are provided as a Source Data file.
