# Supplementary material for: The paraventricular thalamus provides a polysynaptic brake on limbic CRF neurons to sex-dependently blunt binge alcohol drinking and avoidance behavior in mice
Source: Nat Commun. 2021 Aug 23;12:5080. doi: 10.1038/s41467-021-25368-y (PMC8382748; doi:10.1038/s41467-021-25368-y)
Supplement: Supplementary file 2 — Reporting Summary [file 41467_2021_25368_MOESM2_ESM.pdf]

nature research

Corresponding author(s): Kristen E. Pleil

Last updated by author(s): Jul 17, 2021

Reporting Summary

Nature Research wishes to improve the reproducibility of the work that we publish. This form provides structure for consistency and transparency in reporting. For further information on Nature Research policies, see our Editorial Policies and the Editorial Policy Checklist.

Statistics

For all statistical analyses, confirm that the following items are present in the figure legend, table legend, main text, or Methods section.

n/a	Confirmed	
☐	☒	The exact sample size (*n*) for each experimental group/condition, given as a discrete number and unit of measurement
☐	☒	A statement on whether measurements were taken from distinct samples or whether the same sample was measured repeatedly
☐	☒	The statistical test(s) used AND whether they are one- or two-sided<br>*Only common tests should be described solely by name; describe more complex techniques in the Methods section.*
☐	☒	A description of all covariates tested
☐	☒	A description of any assumptions or corrections, such as tests of normality and adjustment for multiple comparisons
☐	☒	A full description of the statistical parameters including central tendency (e.g. means) or other basic estimates (e.g. regression coefficient) AND variation (e.g. standard deviation) or associated estimates of uncertainty (e.g. confidence intervals)
☐	☒	For null hypothesis testing, the test statistic (e.g. F, t, r) with confidence intervals, effect sizes, degrees of freedom and P value noted<br>*Give P values as exact values whenever suitable.*
☒	☐	For Bayesian analysis, information on the choice of priors and Markov chain Monte Carlo settings
☒	☐	For hierarchical and complex designs, identification of the appropriate level for tests and full reporting of outcomes
☐	☒	Estimates of effect sizes (e.g. Cohen's *d*, Pearson's *r*), indicating how they were calculated

Our web collection on statistics for biologists contains articles on many of the points above.

Software and code

Policy information about availability of computer code

Data collection	Slice electrophysiology data were collected using pClamp 10 , slice calcium imaging using MicroManager 1.4 with OptiMos camera, behavior with SMART 2.0 (Pan labs) and Ethovision 10 & 11 (Noldus), and images with a Zeiss LSM 880 confocal microscope.
Data analysis	Slice electrophysiology data were analyzed using Clampfit 10, behavior analyzed with Ethovision 10 & 11, tracing confocal images quantified with ImageJ 1.50 and overlaid using custom MATLAB code using version 9.4 (adapted from Beyeler et al., 2018 and available on Figshare), and slice calcium imaging data with custom MATLAB code (also available on Figshare). Statistics were performed in GraphPad Prism 8.

For manuscripts utilizing custom algorithms or software that are central to the research but not yet described in published literature, software must be made available to editors and reviewers. We strongly encourage code deposition in a community repository (e.g. GitHub). See the Nature Research guidelines for submitting code & software for further information.

Data

Policy information about availability of data

All manuscripts must include a data availability statement. This statement should provide the following information, where applicable:

- Accession codes, unique identifiers, or web links for publicly available datasets
- A list of figures that have associated raw data
- A description of any restrictions on data availability

Source data for all figures is included as a Source Data file. All data that support the findings of this study are deposited on Figshare (https://doi.org/10.6084/m9.figshare.c.5517420.v2).

Field-specific reporting

Please select the one below that is the best fit for your research. If you are not sure, read the appropriate sections before making your selection.

- [x] Life sciences
- [ ] Behavioural & social sciences
- [ ] Ecological, evolutionary & environmental sciences

For a reference copy of the document with all sections, see nature.com/documents/nr-reporting-summary-flat.pdf

Life sciences study design

All studies must disclose on these points even when the disclosure is negative.

Sample size	Because there are few papers in the literature using this experimental design (electrophysiology with SABV, three-way ANOVA with repeated measure), sample sizes required were estimated based on our previously published works in males (Pleil et el., 2016: Rinker et al., 2016; Pleil et al, 2016) showing that Ns of 6+ per group are needed for ChR2 and DREADD experiments, and ns of 10+ cells to detect an effect size of 1 with alpha = 0.05. ~30% more mice were used per group for experiments requiring stereotaxic injections to account for low virus expression in target regions. Additionally, checking distributions of the data for each measurement showed that data adhered to the assumption of parametric analyses such as ANOVA (e.g., data for all groups were from the same normal distribution, an example of which is presented in Supplementary Fig. 8), adding power for the overall and post hoc analyses that ensures ability to measure differences when present.
Data exclusions	As described in the Methods, all data sets were checked for their distributions and analyzed in the space (raw, log) in which they were distributed. All electrophysiology data were normally distributed in log space based on QQplots and outlier tests, resulting in the exclusion of only a few cells for synaptic drive measurements (7c, S1c) and sPSC kinetics (S11). Electrophysiological properties were distributed in normal space, resulting in 0-2 outliers/group for Ih (S9, S11). Outliers are shown in Source Data file. Mice used in DREADD behavior and with ChR2 were excluded if there was no viral expression (misses) or if they met criteria for injection-induced suppression of behavior (compared to no-injection baseline) as described in the Methods. Exclusion criteria for all experiments were the same in control and experimental mice, and exclusions are reported.
Replication	Electrophysiology and multiplexed DREADD behavior experiments were performed in three or more distinct cohorts with all groups evenly represented in each to ensure consistent effects. A first cohort of multiplexed DREADD mice (not included) showed similar effects to the cohorts represented here, however more than half of the mice displayed viral expression following immunohistochemistry that was considered broader than the discrete loci targeted. The same virus lots showed problematic expression in experiments unrelated to this manuscript, so all behavior data using those lots has been considered unreliable and excluded from this and other manuscripts. Two subsequent cohorts were run using a new virus lot that showed appropriate expression in pilot testing and in post hoc virus expression analysis of mice presented in this paper. Results across behavior cohorts were similar. Slice electrophysiology experiments were replicated across multiple cohorts for alcohol-induced plasticity and depending on the specific measures, and converging experimental approaches were further employed to replicate key findings. For example, mPSCs confirmed sPSC results, and these matched oEPSC amplitude results that were confirmed with the PPR experiment.
Randomization	Control vs. experimental virus injections were pseudo-randomized, such that in general, half of each cage used received the control virus and half the experimental virus, with mice in each group having balanced age and weight distributions and means.
Blinding	Experimenters were blind to conditions (except sex, which could not be concealed) for all experiments. Labeled mouse number was not consistently related to condition and unknown to the experimenter, who recorded analyzed the data prior to decoding whenever possible. CON and experimental mice in behavior were run in random order on the same day in order to avoid order/time of day effects, and slice electrophysiology was performed by the same experimenter on 1 CON and 1 EtOH mouse of the same sex per day (or alternating in pseudo-random order in consecutive days). Data were analyzed in the order collected to also avoid any bias in analysis.

Reporting for specific materials, systems and methods

We require information from authors about some types of materials, experimental systems and methods used in many studies. Here, indicate whether each material, system or method listed is relevant to your study. If you are not sure if a list item applies to your research, read the appropriate section before selecting a response.

Materials & experimental systems

n/a	Involved in the study	
☐	☒	Antibodies
☒	☐	Eukaryotic cell lines
☒	☐	Palaeontology and archaeology
☐	☒	Animals and other organisms
☒	☐	Human research participants
☒	☐	Clinical data
☒	☐	Dual use research of concern

Methods

n/a	Involved in the study	
☒	☐	ChIP-seq
☒	☐	Flow cytometry
☒	☐	MRI-based neuroimaging

Antibodies

Antibodies used	anti-DsRed polyclonal in rabbit (Takara Bio, Inc., cat # 632496, lot # 1805060); Alexa Fluor 594 donkey anti-rabbit (Jackson ImmunoResearch, cat # 711-585-152, lot # 133200)
Validation	Antibodies are validated by the companies we purchased from. See web page https://www.takarabio.com/products/antibodies-and-elisa/fluorescent-protein-antibodies/red-fluorescent-protein-antibodies and certificate of analysis: https://www.takarabio.com/assets/documents/Certificate%20of%20Analysis/632496-101717.pdf. We also validated using positive and negative controls in our lab using control mice (including those with other fluorophores to ensure specificity) prior to using our immuno protocols for virus expression confirmation in this paper.

Animals and other organisms

Policy information about studies involving animals; ARRIVE guidelines recommended for reporting animal research

Laboratory animals	All mice used were young adult mice (8-10 weeks at start of study) on a C57BL/6J background strain, including wildtypes purchased from Jackson Laboratories at 6-8 weeks of age and those bred in our lab (VGLUT2-ires-Cre and CRF-ires-Cre from Bradford Lowell and floxed Ai9 mice from Jax).
Wild animals	The study did not involve wild animals.
Field-collected samples	The study did not include samples collected from the field.
Ethics oversight	All experiments performed were approved by the Institutional Animal Care and Use Committees of Weill Cornell Medicine and the University of North Carolina-Chapel Hill.

Note that full information on the approval of the study protocol must also be provided in the manuscript.
